# The Emerging Role of the Gut Microbiome in Cardiovascular Disease: Current Knowledge and Perspectives

**DOI:** 10.3390/biomedicines10050948

**Published:** 2022-04-20

**Authors:** Panagiotis D. Papadopoulos, Christina Tsigalou, Pipitsa N. Valsamaki, Theocharis G. Konstantinidis, Chrysoula Voidarou, Eugenia Bezirtzoglou

**Affiliations:** 1 Master Programme Food, Nutrition and Microbiome, Department of Medicine, Democritus University of Thrace, 68100 Alexandroupolis, Greece; panapapa19942511@gmail.com (P.D.P.); empezirt@med.duth.gr (E.B.); 2 Laboratory of Microbiology, Department of Medicine, Democritus University of Thrace, 68100 Alexandroupolis, Greece; 3 Nuclear Medicine Department, Medical School, Democritus University of Thrace, 68100 Alexandroupolis, Greece; pivalsam@med.duth.gr; 4 Blood Transfusion Center, University General Hospital of Alexandroupolis, 68100 Alexandroupolis, Greece; tkonsta@med.duth.gr; 5 Department of Agriculture, University of Ioannina, 47132 Arta, Greece; xvoidarou@uoi.gr; 6 Laboratory of Hygiene and Environmental Protection, Department of Medicine, Democritus University of Thrace, 68100 Alexandroupolis, Greece

**Keywords:** microbiome, cardiovascular disease, gut dysbiosis, CVD, bacterial metabolites, TMAO

## Abstract

The collection of normally non-pathogenic microorganisms that mainly inhabit our gut lumen shapes our health in many ways. Structural and functional perturbations in the gut microbial pool, known as “dysbiosis”, have been proven to play a vital role in the pathophysiology of several diseases, including cardiovascular disease (CVD). Although therapeutic regimes are available to treat this group of diseases, they have long been the main cause of mortality and morbidity worldwide. While age, sex, genetics, diet, tobacco use, and alcohol consumption are major contributors (World Health Organization, 2018), they cannot explain all of the consequences of CVD. In addition to the abovementioned traditional risk factors, the constant search for novel preventative and curative tools has shed light on the involvement of gut bacteria and their metabolites in the pathogenesis of CVD. In this narrative review, we will discuss the established interconnections between the gut microbiota and CVD, as well as the plausible therapeutic perspectives.

## 1. Introduction

As “everything touches everything else”—the so-called “connectome”—the human body is ceaselessly exposed to the environment since its creation, and subsequently trillions of commensal microorganisms (approximately 10^13^–10^15^) subsequently colonize the human body [1,2]. This astonishingly diverse ecosystem, described by the term “microbiome”, consists of the combined genetic capacities of bacteria, viruses, protozoa, and fungi and is described by the term “microbiome”. It is well established that the microbiota is composed of 100 times more unique genes than those of human origin that are codified, and exerts myriad physiological functions [3]. Most of these microbes dwell in the human gastrointestinal tract, and are termed “gut microbiota”.

While the gut ecological community is characterized by great diversity, in the absence of disease its composition remains relatively stable. The normally dominant phyla are *Firmicutes* and *Bacteroidetes*, which account for 90% of bacterial species inhabiting the human gut [4]. *Proteobacteria*, *Actinobacteria*, *Cyanobacteria*, *Fusobacteria*, and *Verrucomicrobia* comprise most of the remaining percentage [5].

The unique microbial “fingerprint” every human possesses is formed by numerous factors from our very earliest days. The mode of delivery is one of the abovementioned impactful elements. In the event of caesarian section, microbes are transferred from the hospital environment and the medical staff to the newborn [6], while in a normal delivery through the genital tract the maternal vaginal flora play a leading role [7]. Feeding style (breastfeeding or formula) and gestational age have also been linked to certain microbial “signatures”. During the later stages of life, host genome, sex, personal habits, lifestyle, geography, air pollution, infectious conditions, medicament intake (especially antibiotics), vaccinations, stress levels, and hormonal status determine the gut microflora [8,9,10,11,12].

The collection of normally non-pathogenic microorganisms that mainly inhabit our gut lumen shapes our health in many ways. From the neonatal period onwards, the gut microbiota determines the formation of the intestinal architecture [13], and regulates the mechanisms of metabolism and local immunity [14,15,16]. Structural and functional perturbations in the gut microbial pool, known as “dysbiosis”, have been proven to play a vital role in the pathophysiology of several conditions, including inflammatory bowel disease (IBD), obesity, autism, cancer, and cardiovascular disease (CVD) [17,18,19].

The term CVD stands for a group of diseases with great heterogeneity and numerous different manifestations. Atherosclerosis (and thrombosis), cerebrovascular disease, hypertension, heart failure, atrial fibrillation, and myocardial fibrosis are some pathological cardiac-related events that play havoc with human lives on a daily basis. Although therapeutic regimes are available to deal with this group of diseases, they have long contributed to the main causes of mortality and morbidity worldwide [20]. Specifically, according to the WHO, an estimated 17.9 million people died from CVD in 2019, representing 32% of all global deaths. Of these deaths, 85% were due to heart attack and stroke. While age, sex, genetics, diet, tobacco, and alcohol abuse are major contributors (World Health Organization, 2018), they cannot explain all of the consequences of CVD [21]. In addition to the aforementioned classic risk factors, there are several lines of evidence suggesting the participation of non-traditional agents in CVD manifestations, such as chronic kidney disease (CKD), rheumatoid arthritis (RA), human immunodeficiency virus (HIV), history of malignancy, and more specific indices (e.g., the ankle-brachial index, hsCRP, coronary artery calcium scoring). This constant search for novel preventative and curative tools has shed light on the involvement of gut bacteria and their metabolites in the pathogenesis of CVD [22,23].

The involvement of microbiome composition in CVD development and progression seems to have been intensely examined by a number of experimental studies, but is beyond the scope of this review. The objectives of this narrative review are to discuss the established interconnections between gut microbiota and CVD derived from research in humans, as well as the plausible therapeutic perspectives. All of the pieces of information were retrieved between June 2021 and January 2022 from the databases MEDLINE/PubMed, from peer-reviewed articles and systematic reviews published in English since 1982. Nonetheless, the majority of articles concerning the principal concept included recent publications. The main keywords were the following: cardiovascular disease, gut microbiome, gut dysbiosis, bacterial metabolites, TMAO. In the end, noteworthy references that appeared in the selected articles were also considered and surveyed. This review is divided by subheadings to create a concise and comprehensive presentation of the subject.

### 1.1. Indirect Association of Alterations in the Intestinal Microbiota and Significant Risk Factors for CVD

Distinct alterations in the configuration of the intestinal microbiota, videlicet, “dysbiosis”, have been closely linked to certain CVD phenotypes. Admittedly, there is no such a thing as a pathogenic microbial “signature”, but the metagenomic era has led to a wealth of associations between an imbalanced gene pool and adverse cardiovascular events.

#### 1.1.1. Diabetes Mellitus

Type II diabetes mellitus (T2DM) ranks unquestionably high on the list of risk factors for CVD, and is accompanied by certain gut microbial perturbations. Specifically, patients with T2DM present reduced abundance of bacterial genera such as *Bifidobacterium*, *Faecalibacterium*, *Bacteroides*, *Akkermansia*, and *Roseburia*. The latter three impede the inflammation process, as they promote the production of anti-inflammatory cytokines and chemokines (e.g., IL-10 and 22, TGF-β) while inhibiting the formation of the pro-inflammatory ones (e.g., IL-1β, IL-8, IL-16 and 17, CD36, IFN-γ, NF-κΒ, monocyte chemoattractant protein-1, intercellular adhesion molecule-1) and C-reactive protein. The lower concentrations of the genera *Bacteroides* and *Akkermansia* can lead to underexpression of tight junctions’ genes, increased “leaky gut”, and, consequently, endotoxemia [24]. Additionally, the lower levels of the butyrate-producing *Roseburia intestinalis* and *Faecalibacterium prausnitzii* have been noted to dysregulate the metabolism of fatty acids, creating oxidative stress and promoting cardiometabolic adverse manifestations [25,26].

The other side of the coin of dysbiosis in DM is its positive association with bacteria from the genera *Ruminococcus*, *Fusobacterium*, and the phylum *Firmicutes*. These microbes flare up the inflammation process by producing a “reservoir” of inflammation-inducing cytokines [27].

#### 1.1.2. Hypertension

Persistently elevated blood pressure (BP) is one of the most prevalent factors that lead to CVD worldwide, and the leading cause of disability and death in Western societies [28]. Numerous experimental projects have already confirmed the associations between gut microbiome composition (significantly lower gene richness and α-diversity) and BP regulation. Specifically, the vast majority of these studies have confirmed that hypertensive individuals have a significantly higher (up to fivefold) *Firmicutes-to-Bacteroides* ratio compared to normotensive ones [29]. Furthermore, in the presence of hypertension, the intestinal microbiota is dominated by lactate-producing genera (e.g., *Streptococcus* and *Turicibacter*), while short-chain-fatty-acid-producing ones appear to be decreased (such as *Akkermansia*, *Bacteroides*, and *Clostridiaceae*) [30,31]. The importance of short-chain fatty acids (SCFAs) lies in their effects on renal olfactory receptor (Olfr) 78 and G-protein-coupled receptor (Gpr) 41, which are expressed in the vascular smooth muscle cells and regulate vasodilation [32,33]. An up-to-date Brazilian study added a new perspective to the abovementioned data, correlating the pathologically high BP with an abnormally high TNF-α/IFN-γ ratio [34].

While all of these differences indicate a clear microbial aspect of hypertension, the demonstration of causal relationships is a much bigger challenge. Towards this direction, only a few studies have been conducted, proving that the phenotype of heightened BP is transferable from diseased individuals to germ-free ones via fecal microbial transplantation [35,36].

#### 1.1.3. Hypercholesterolemia

High serum cholesterol, known as hypercholesterolemia, is a long-standing contributor to CVD. The build-up of cholesterol in the arterial wall creates plaques that can lead to atherosclerosis [37]. In the human body, the cholesterol homeostasis mechanism represents a multidimensional system. Its main axes include de novo synthesis of cholesterol [38], liver-located conversion into bile, and intestinal absorption [39,40]. The gut lumen for its part plays an eminent role in these processes and, by extension, in the pathogenesis of CVD. Firstly, there are gut bacterial genera with bile salt hydrolase (BSH) activity that greatly influence the sophisticated mechanism of bile acid production [41]. Specifically, *Lactobacillus*, *Clostridium*, *Listeria*, *Bifidobacterium*, and some members of *Bacteroides* deconjugate the primary bile acids to form secondary ones [42]. As a result, in the case of gut dysbiosis, secondary bile acids can be reduced, leading to an abnormal accumulation of primary bile acids, downregulation of the bile acid production mechanism (FXR–TGR5 pathway) [43,44] and, thus, heightened cholesterol. Another linkage between the gut microbiota and lipid metabolism is the conversion of cholesterol to coprostanol—a process that is carried out by certain bacterial strains, mainly from the genera *Lactobacillus* and *Eubacterium* [45,46]. Alterations in the abundance of these cholesterol-reducing microorganisms (possessing the reductase enzyme) can impede the elimination of cholesterol from the body. The lower concentrations of SCFA-producing bacteria, which we have already discussed earlier, can also be associated with insulin-mediated fat agglomeration due to the activation of G-protein coupled receptor 43 (GPR43) [47].

#### 1.1.4. Obesity/Metabolic Syndrome and Lifestyle

Obesity and metabolic syndrome comprise a global pandemic that goes hand in hand with the ongoing outburst of CVD cases [48,49]. Gut dysbiosis is considered to be an impactful pro-inflammatory factor with a major effect on the aforementioned health conditions [50,51]. Particularly, in both cases, a certain recurring microbial pattern has been detected at several ages, with higher abundance of *Firmicutes* and lower abundance of *Bacteroidetes* (up to 50% decrease) [52,53]. Fecal microbiota transplantation (FMT) studies between lean and obese mice have proven that both phenotypes are highly transmissible from the one group to the other, suggesting the driving force of the gut microbiome [54]. *Enterobacter cloacae*, for instance, when isolated from obese human subjects and inoculated in germ-free mice, led to obesity and insulin resistance [55]. The “-omics” technologies, studying large sets of biological molecules, have shed even more light on the obesogenic gut microbiome. Specifically, *Akkermansia muciniphila*, *Clostridium bartlettii*, and *Bifidobacteria* have been negatively correlated with high-fat-diet-induced obesity and metabolic complications [46,56]. These SCFA-producing bacteria are vital for the maintenance of the intestinal epithelial integrity, as well as prevention of bacterial translocation into the bloodstream, and subsequent endotoxemia [57].

We should not overlook the fact that both obesity and metabolic syndrome are health issues affected to a great degree by lifestyle factors that must also be taken into account. The level of physical activity is constantly gaining more and more popularity. Interestingly, a recent study shows that a sedentary way of life has the exact same impact on the gut microbiome as obesity and MetS [58].

#### 1.1.5. Immune System Implication

The gut microbiota can also induce the onset of CVD via the manipulation of host immune responses. Several components of the cascade of innate immunity are greatly affected by our internal bacterial milieu. For example, individuals with low microbial gene richness (LGC) simultaneously have high white blood cell (WBC) counts and CRP levels—parameters that establish a pro-inflammatory status, promoting the manifestation of CVD [59,60]. Our microflora also participates in the expression of pattern recognition receptors (PRRs) in the intestinal epithelium [such as toll-like receptors (TLRs) and NOD/CARD proteins] and affects regulatory T (Treg) cells [61]. Another piece of the “puzzle” engages oxidized LDL (oxLDL), whose engulfment by macrophages activates foam and T cells, and leads to a reservoir of inflammatory cytokines (e.g., TNF-a, IL-1β, IL-6, IL-18, IL-37) [62]. Specific host immune responses that participate in CVD and have been elucidated, also include the Th17 response [63] and IL-22 pathway [64].

It is worth mentioning that gut-microbiome-derived TMAO can also activate the immunological arsenal of the TXNIP–NLRP3 inflammasome pathway, which is tightly linked to CVD [65,66]. Under certain conditions, even the otherwise beneficial SCFAs have been correlated with TLR4-mediated inflammatory response [67]. Finally, the “leaky gut” concept also proves the immense effect of the gut microbiome on the host immune system. The impaired gut barrier integrity leads lipopolysaccharides and other bacterial wall products to enter the circulation, orchestrating a pro-inflammatory state [68,69].

#### 1.1.6. Gut Metabolites

In order to achieve further, deeper understanding of the pathogenetic mechanisms between the gut microbiome and CVD, it is crucial to understand the importance of the microbial metabolites accompanying any bacterial signature. All of these components constitute a community of interacting biological entities, termed metaorganism. TMAO, first and foremost, is a metaorganismal metabolite with the potential to become a non-traditional CVD biomarker. Dietary precursors—mainly including choline, phosphatidylcholine, and carnitine—are converted by specific bacterial TMA lyases to TMA [70,71,72], which is turned into TMAO by host hepatic flavin-containing monooxygenases, such as FMO3 [73]. TMAO has been positively correlated with enhanced atherosclerosis [74,75] and cholesterol-laden macrophage foam cell formation [76], platelet hyper-reactivity (through calcium release), and increased thrombosis potential, vascular inflammation, and inflammasome activation [77,78,79]. Consequently, numerous large-scale clinical cohorts have established the vital involvement of TMAO in many CVD phenotypes, such as coronary artery disease (CAD) [80], heart attack and ischemic stroke [81,82], heart failure [83,84], acute coronary syndrome [80], and peripheral artery disease [85]. Bile acids are also responsible for the modulation of certain diseases, as we have already discussed above. An altered bile acid pool can wreak havoc on the host metabolism and trigger an inflammatory response linked to hypercholesterolemia, insulin resistance [86], atherosclerosis [87], and heart failure [88,89]. For a complete approach to gut metabolites, it is fundamental to understand the importance of SCFAs. SCFA-mediated mechanisms exert a plethora of actions, such as BP regulation [33,90], prevention of fat accumulation [47], anti-inflammatory effects [91], protection of gut barrier coherence [57], energy expenditure, and control of colonic pH [92]. It poses no surprise that imbalances regarding the SCFA-producing bacteria can lead to various diseased states, including hypertension, obesity, atherosclerosis, and endotoxemia.

### 1.2. Direct Effects of Dysbiosis on Cardiovascular Health

Gut dysbiosis undeniably has a massive indirect impact on several CVD risk factors, including T2DM, hypertension, hypercholesterolemia and obesity. Furthermore, a disorganized gut microbial community can directly undermine cardiovascular functionality. Atheromatosis, CAD, coronary artery disease, chronic heart failure, and atrial fibrillation are conditions facilitated by the oxidative stress and vascular inflammation exerted by gut dysbiosis, and are discussed in further detail below.

#### 1.2.1. Atheromatosis and CAD

Early studies by Koren et al. indicated the influence of the gut microbiome in the process of plaque formation, as microbial genetic material was retrieved from atherosclerotic deposits [93]. Recent metagenomic analyses of carotid thrombi validate the suggestion of bacterial translocation into the bloodstream and subsequent lodgment in clots [94]. Both microbial translocation and gut microbiota dysbiosis have been linked to CAD by the induction of lipid metabolism alteration and systemic vascular inflammation [95]. The direct impact of dysbiosis on CVD is also exerted by the generation of detrimental metabolites. Particularly, it comes as no surprise that TMAO plays a prominent role in atherosclerotic plaque enhancement, exacerbating the vascular wall’s inflammatory reactions, promoting reactive oxygen species production, and inhibiting cholesterol reverse transport [65]. Additionally, a positive correlation between plasma concentration of TMAO and plaque size has also been confirmed [72]. To date, a group of infectious agents (e.g., *Helicobacter pylori*, *Hepatitis C virus*, *Cytomegalovirus*, *Chlamydia pneumoniae*) and opportunistic pathogens (such as *Enterobacter*, *Desulfovibrio*, *Megasphaera*, and *Oscillibacter*) also seem to promote the atherosclerotic process [96].

#### 1.2.2. Chronic Heart Failure

Chronic heart failure (CHF) is another CVD that is characterized by a low cardiac output or an increased ventricular filling pressure. The manifestation of CHF can be attributed to a certain degree to the gut microbiota. Significant differences have been established between the microbiomes of CHF patients and those of healthy controls, such as the high abundance of bacterial species linked to TMAO production (including *Anaerococcus hydrogenalis*, *Clostridium asparagiforme*, *Clostridium sporogenes*, *Edwardsiella tarda*, *Proteus penneri*, and *Providencia rettgeri*) [97]. Further evidence of the direct effect of the gut microflora on the mechanisms of CHF is the faulty intestinal barrier function. The disease severity has been positively correlated with gut wall permeability [98], as the compromised blood flow to the lumen leads to the disruption of its integrity and the subsequent establishment of low-grade systemic inflammation [99]. The leaky gut allows different microbial components—such as LPS and peptidoglycan—to reach the circulation and initiate an inflammatory response, as described earlier. The importance of the dissociated endotoxins in the CHF was also obvious in the case of individuals with edematous heart failure, who had much more prevalent endotoxemia compared to non-edematous controls [100].

#### 1.2.3. Atrial Fibrillation

Atrial fibrillation is the most common cardiac arrhythmia, affecting more than 37 million people worldwide [101]. Despite the existing knowledge gaps, there are several lines of evidence suggesting the AF-promoting properties that the intestinal microbiome possesses. Recent observational studies have outlined the gut microbial profiles of AF patients, which are described by a dramatically high *Firmicutes/Bacteroidetes* ratio, the aftereffects of the overgrowth of harmful bacteria (such as *Streptococcus, Enterococcus*, and *Escherichia coli*), and the lower abundance of commensals (e.g., *Faecalibacterium*, *Prevotella*) [102,103]. These microbial shifts are also accompanied by alterations in metabolites. More specifically, clinical data have correlated AF with increased TMAO levels [104], but in an inconclusive way [105]. Circulating bile acids [106], SCFAs, and indoxyl sulfate [107] have also been confirmed as potential contributors to the pathogenesis of AF. The most potent evidence for a direct impact of gut dysbiosis on AF lies in the findings of an up-to-date and elegant study [108]. Zhang et al. demonstrated the involvement of age-associated gut imbalances in the onset of AF through the high serological concentrations of LPS and glucose (due to the increased intestinal permeability) in combination with the overactivation of the atrial NLRP3 inflammasome. Critical structural (atrial fibrosis) and functional (reentry-promoting abbreviation of the atrial action potential, higher frequency of spontaneous diastolic sarcoplasmic reticulum Ca2+ releases) changes can be attributed to this enhanced cardiomyocyte inflammatory signaling, determining the features of the disease [109,110].

## 2. Current Therapeutic Interventions and Future Perspectives in the Context of Personalized Medicine

### 2.1. Different Diets and Their Role in the Development of CVD

Diet plays the most prominent role among the environmental exposures that shape the human gut microbiome and, consequently, significantly affect the development of non-communicable diseases such as CVD. A plethora of studies have investigated the impact of established dietary regimes on the manifestation of the aforementioned adverse phenotypes, with solid outcomes. The Western pattern diets have been in the spotlight, being characterized by high intake of saturated fats, salt, and sucrose, as well as low intake of fiber, to promote gut dysbiosis and CVD later on [111,112]. Particularly, this modern dietary path is connected to a disturbed gut microbiome with decreased microbial richness, high *Firmicutes/Bacteroidetes ratio*, and increased concentrations of certain pro-inflammatory bacteria, such as Escherichia coli [113,114,115]. Beneficial bacterial lineages (such as *Lactobacillus murinus*, *Bilophila wadsworthia*, and *Akkermansia muciniphila*) are depleted, setting the host’s immune system up for detrimental cardiovascular effects [116,117].

By contrast, the Mediterranean diet has a tremendously positive impact on the microbiome ecosystem network and the prevention of CVD. High intake of fiber, and a balanced ratio of omega-6/omega-3 essential fatty acids, vitamins, and natural antioxidants compose the macronutrient profile of the MedDiet [118]. Adherence to this dietary pathway has been linked to a higher proportion of *Bacteroidetes* (and a lower *F/B ratio* as a result) [119], as well as generally of other fiber-degrading microorganisms, leading to increased SCFA levels and lower inflammation (through the G-protein-coupled mechanism we described earlier) [32,120]. The cascade of SCFA production is also activated by the ω-3 polyunsaturated fatty acids (PUFAs) [121]. Another vital byproduct of plant-based diets (such as the MedDiet) is the significantly lower concentration of TMAO [122]—an immune system modulator causally linked to several CVD types [123,124,125].

While a complete analysis of this topic is beyond the scope of this review, there are some other critical points that should not be overlooked. Firstly, the development of CVD is a multifactorial process, so trying to investigate the individual effects of certain macronutrients on it is a big challenge. On top of that, diets such as the MedDiet and their consequences are a matter of a whole lifestyle, and not only of dietary habits [126].

With that being said, the established striking connection between the onset of CVD and an imbalanced gut microbiota comes as no surprise. Consequently, the gut microflora is a parameter that should be taken into consideration in the various CVD therapeutic strategies in order to amplify treatment outcomes.

### 2.2. Pharmacological Therapy

Drug therapy is the most conventional treatment “weapon” in the battle against CVD. HMG-CoA reductase inhibitors—the most well-known statins [127]—control hypercholesterolemia and reduce the risk of CAD by regulating the rate-limiting enzyme in cholesterol synthesis [128]. Interestingly, the three most commonly used statins—simvastatin, rosuvastatin, and atorvastatin—seem to exert their function by determining the composition of the gut microbiota [129]. Khan, T.J. et al. proved that patients treated with atorvastatin displayed an anti-inflammatory gut microbial profile compared to non-treated individuals [130]. Another very recent study also validated the gut-microbiome-mediated action of statins [131]. The inefficiency of statins in non-LDL cholesterol cases, in combination with their side effects [132], has led to complementary drug substances with different targeting. Ezetimibe is a typical example, which sufficiently inhibits intestinal LDL-cholesterol absorption by blocking hepatic NPC1L1 [133,134]. Similarly to statins, however, ezetimibe’s prescription could also backfire, leading to adverse events such as increase in cholesterol and the formation of gallstones, and complicating the treatment [135,136].

Antibiotics are also widely used in clinical practice, with great ambiguity. In experimental animal models of hypertension, the administration of broad-spectrum antibiotics such as minocycline, neomycin, vancomycin, ampicillin, and gentamycin lowered the arterial pressure by restoring the composition of the gut microbiome [17,31]. However, on the other hand, the other side of the coin is that antibiotics constitute a poor long-term therapeutic intervention, as they lack clinical validity [137,138] and contribute massively to the development of antibiotic resistance. Moreover, the more or less unpredictable effects of these drugs on the gut bacterial community provide fertile ground for opportunistic infections.

Antihypertensive medication—such as beta-blockers, angiotensin II receptor blockers (ARBs), and angiotensin-converting enzyme (ACE) inhibitors—may also alter intestinal homeostasis to exert efficacy. Thoroughly examined drugs include candesartan [139] and captopril [140], which regulate the blood pressure by restoring the gut lumen’s bacterial communities and integrity, respectively. Nifedipine is another antihypertensive drug that modulates the intestinal metabolic milieu, inducing the production of 3-(3-hydroxyphenyl) propionate (3-HPP) and reducing the levels of deoxycorticosterone [141,142]. Aspirin, a non-steroidal anti-inflammatory drug (NSAID), is also an integral part of the established methodology applied to cardiovascular and cerebrovascular diseases. The administration of aspirin seems to induce positive microbiome shifts, enriching protective species such as Bacteroides, Prevotella, Ruminococcaceae, and Barnesiella [143], accompanied by relatively low intestinal wall damage [144]. To complete the approach of drugging the microbiome in the context of CVD, the antidiabetic drug metformin should also be mentioned. Multiple studies have shed light on the pleiotropic cardioprotective effects of metformin, associating them with the intestinal bile acid pool, the production of SCFAs, and critical regulators of the intestinal immune system, among others [145,146].

### 2.3. FMT and Pre-/Probiotics

Despite the pharmaceutical “arsenal” for the treatment of CVD, clinical outcomes are often far from desirable. This makes the integration of new strategies in the clinical praxis an issue of great urgency. The administration of prebiotics, probiotics, and fecal microbiota transplantation (FMT) are typical examples in this direction.

FMT is a relatively straightforward therapeutic method that manipulates the host gut microflora in order to restore gut eubiosis and improve the progress of certain diseases. Metabolic syndrome—a major CVD risk factor—has been investigated in several human FMT studies. While some of them failed to connect FMT with symptom reduction [147], the setup of some technical parameters (such as FMT material, route of administration, and colon preparation) in others led to increased FMT-induced insulin sensitivity [148]. Recently, an experimental animal model proved that the gut microbiota transfer from healthy donors to diabetic rats alleviated the manifestations attributed to T2DM [149].

Despite the several lines of evidence that a disturbed gut microbial ecosystem is a critical part of CVD pathophysiology, just a small portion of the currently available literature explores the therapeutic potential of FMT in this context. Murine models showed that transplantation of healthy stools can ameliorate both myocardial damage [150] and hypertension [151]. As far as the human studies are concerned, the one and only project examined the impact of FMT from vegan donors on vascular inflammation and TMAO levels. After the intervention, compositional changes were observed in the recipients’ gut microbiome, but without eliciting alterations in either TMAO production or vascular inflammation [152].

Prebiotics (substances selectively utilized by the host’s microorganisms, conferring health benefits) [153] and probiotics (live microorganisms that when administered in adequate amounts may improve the host’s health) [154] have been the focus of interest of more and more studies, whose outcomes highlight their therapeutic potential. Core probiotic genera such as *Lactobacillus*, *Bifidobacterium*, *Saccharomyces*, *Streptococcus*, and *Enterococcus* have been found to improve several CVD risk factors and phenotypes. Starting from T2DM, Khalili et al. showed that the provision of *Lactobacillus casei* 01 to diabetic patients led to boosted glycemic control [155]. Similarly, treatment of diabetic rats with *Lactobacillus paracasei* NL 41 increased their insulin sensitivity and conferred β-cell protection—a critical cardioprotective marker [156,157]. However, it should be noted that according to an up-to-date meta-analysis, any recorded improvement (by probiotics, prebiotics, or synbiotics) was of low magnitude [158].

Probiotics have also been evaluated for their pressure-lowering action. Treatment with a 1:1 mixture of *L. coryniformis* CECT5711 and *L. gasseri* CECT5714 (K8/LC9) improved endothelial function and blood pressure regulation by reducing the vascular inflammatory response [159]. Co-supplementation of *Lactobacillus rhamnosus* G and prebiotic inulin also seems to lead to a subsided inflammatory profile in individuals suffering from CAD [160]. In the same study, a promising probiotic mechanism of action came to the fore—that of the inhibition of the TMAO pathway. Moreover, accruing evidence reveals that probiotics also have the potential to decrease cholesterol levels. Particularly, *Akkermansia muciniphila* is of utmost importance, as its provision in human studies improved several clinical parameters [161]. Despite the relatively few studies discussing the effects of prebiotics on CVD, their antioxidant capacities are coming to light. In addition to the abovementioned inulin, very recently a prebiotic complex based on fermented wheat bran was found to exert gut microbiome remodeling properties that ameliorate symptoms of heart failure [162]. Last but not least, yeast β-glucan also seems to have the prebiotic potential for the manipulation of the gut microbiota [163].

Nevertheless, there are plenty of issues that need to be clarified concerning the probiotic strains, the prebiotic substances, the precise dosage, the impact on immunologically vulnerable individuals, and the feasible long-term effects of these biological agents.

### 2.4. New Knowledge and Challenges

New valuable knowledge is consistently accumulated, enhancing our understanding about how the gut microbiota impacts CVD, and creating revolutionary therapeutic tools. As the gut flora is now a well-grounded target for the management of CVD, it is vital that it be approached in the light of innovative methods. One such method is nanomedicine—a multidisciplinary field aiming to develop therapeutic and diagnostic objects that, at least in one dimension, lie between 0.1 and 100 nm [164]. Currently, nanotechnology has already shown immense capacity for the management of CVD. Specially designed nanoparticles, apart from the obvious delivery of conventional drugs, can also shoulder the scavenging of LDL cholesterol [165], the reduction of oxidative stress/local inflammation [166], and the production of anti-inflammatory macrophages [167].

While nanomedicine has already shown vast potential in the context of treating CVD, its possible in the modulation of the gut microbiome to influence the development of CVD is scarce. This flourishing technology could help us to clarify the connection between the gut microbiome and the pathogenesis of CVD, which has been a great challenge thus far. In the concept of protein corona analysis, nanoparticles with an embedded layer of biomolecules in their surface interact with samples of patients. In this way, specific gut microbial patterns and disease mechanisms can be revealed, saving time needed not only for the early diagnosis of CVD, but also for targeted treatment [168,169]. Similar patterns can be created by another nanotechnology-based approach—that of magnetic levitation (Maglev). Plasma proteins are levitated under the influence of superparamagnetic nanoparticles and create protein/biomolecular patterns [170]. Through high-performance data processing, these “signatures” are associated with certain microbial profiles and/or the occurrence of CVD [171].

Apart from the sophisticated diagnostic tools we discussed above, substantial changes should also take place in the ways in which we intervene in the gut microbiota. To that end, and through nanomedicine, nanoparticles could deliver gut microbes capable of increasing HDL and SCFAs, as well as reducing LDL, LPS, TMAO, and pro-inflammatory cytokines. This new aspect of molecule delivery could drastically abate the toxicity of conventional pharmacotherapy [172]. However, constructing such effective and safe particles/vehicles is an obstacle to be overcome. Another major challenge of integrating the gut microbiome into clinical practice is intra- and intervariability among people. As we mentioned before, a number of factors affect the composition of the gut microflora, impeding the identification of biomarkers and therapeutics. Things are becoming even more complicated as a result of a thus-far massively ignored aspect—that of the microbially induced alterations in the drug metabolism [129]. In the direction of precision medicine, further studies about drug pharmacokinetics are needed [173]. Another exciting, yet unexplored, area of investigation is that of the nonlethal microbial inhibitors that are attached to specific metabolic pathways [174].

**Figure 1:**
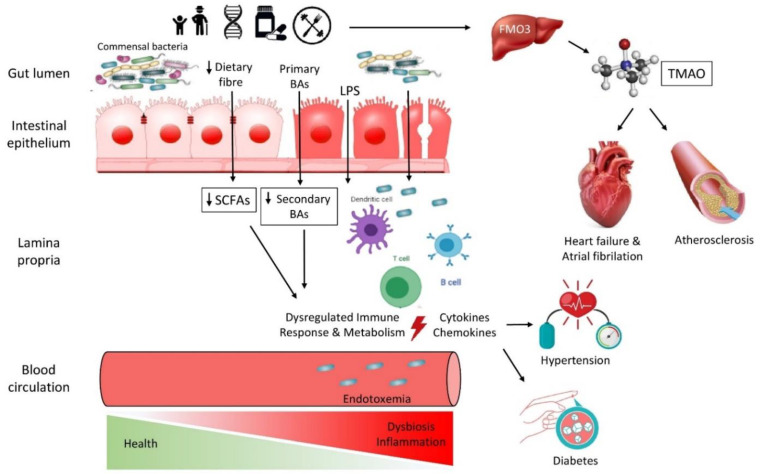
A simplified summary of the interconnections between the gut microbiota and traditional and non-traditional risk factors. TMAO: Trimethylamine-N-oxide, LPS: Lipopolysaccharides, FMO: Flavin-containing monooxygenase, BAs: Bile Acids, SCFAs: Short Chain Fatty Acids.

The prospect of acquiring all of this in-depth knowledge about the gut microbiome–CVD interaction would not be feasible without the backing of rapid technological development. Culture-based strategies have been replaced by next-generation sequencing (NGS) methods and “omics” data that allow us to reach previously unknown aspects of the microbiome [175]. The interpretation of this enormous amount of metagenomic data should lead to reliable pipelines and databases of all of this knowledge. While great progress takes place, there are still a lot of limitations and, as a result, “miles” to cover in order to make the transition from correlation to causality between the microbiome and CVD (Figure 1) [176].

## 3. Conclusions

Summing up, a continuously growing body of experimental data indicates the “dialogue” between the gut microbiome and CVD (Table 1). Gut microbiota dysbiosis is a major determinant for the vast majority of CVD risk factors, as is discussed in this review. Further investigations with the use of state-of-the-art tools will be integral to attaining a more lucid understanding of these complex interconnections. Is an imbalanced gut microbial ecosystem a driving force of CVD, or just a parallel event? Translating this knowledge into high-precision microbiome-mediated CVD strategies will be a game changer, as current treatments seem to be inadequate in many cases. While more and more modernized approaches dominate the field of CVD therapeutics, we should not by any means underestimate the significant impact of more conventional interventions, such as diet and exercise [177].

## Figures and Tables

**Table 1:** Representative clinical trials featuring the interconnection between the gut microbiome and CVD.

	Clinical Trials		
Diseases	Sample (Controls excluded)	Gut Microbiota-related effect	Method
Atheromatosis	100 STEMI patients [178]	Translocation of gut bacteria to the bloodstream	16S rRNA PCR (V4 region)
	4144 older adults [124]	Increased TMAO levels	Stable isotope dilution LC/MS/MS
CAD	29 CAD patients [179]	Increased L-carnitine and TMAO levels	Electrospray ionization LC/MS-8060
	63 non-FH CAD patients [180]	Increased LPS levels and inflammatory cytokines	LBP, ELISA
Chronic Heart Failure	428 HFrEF patients 395 HFpEF patients [181]	Increased TMAO levels—increased HF susceptibility	LC/MS/MS
Heart attack	19 ACS patients [182]	Increased gut leakiness and endotoxemia	L/M ratio, LC/MS, 16S sequencing
Atrial fibrillation	20 psAF patients [183]	Distinctive and progressive alterations in gut microbiome and metabolic structure	LC/MS (+/− ion mode), metagenomic sequencing
	912 AF patients [184]	Increased LPS levels and platelet hyperreactivity	ELISA
	20 psAF—30 PAF patients [182]	Distinctive and progressive alterations in gut microbiome	LC/MS, Whole-metagenome shotgun sequnecing
	117 rheumatic heart disease patients with AF [185]	Increased TMAO levels and thrombus development	Light transmittance aggregometry, LC/MS

STEMI: ST elevation myocardial infarction; psAF: persistent atrial fibrillation; PAF: paroxysmal atrial fibrillation; HFrEF: HF patients with reduced ejection fraction; HFpEF: HF patients with preserved ejection fraction; FH: familial hypercholesterolemia; ACS: acute cardiac syndrome.

## Data Availability

Not applicable.

## Abbreviations

Cardiovascular disease	CVD
Trimethylamine-N-oxide	TMAO
Blood pressure	BP
Bile salt hydrolase	BSH
G protein-coupled receptor	GPR43
Low gene count	LGC
C-reactive protein	CRP
High-sensitivity C-reactive protein	CRP
Ankle brachial index	ABI
Coronary artery calcium	CAC
Tumor necrosis factor alpha	TNF-a
Lipopolysaccharides	LPS
Flavin-containing monooxygenase	FMO
Tissue factor	TF
P-selectin	CD62P
Short-chain fatty acids	SCFAs
Interleukin	IL
Hydroxy-methyl-glutaryl-coenzyme A	HMG-CoA
